# A review of the characteristics of rainfall simulators in soil erosion research studies

**DOI:** 10.1016/j.mex.2023.102506

**Published:** 2023-12-06

**Authors:** Tobias Koch, Peter Chifflard, Peter Aartsma, Kerstin Panten

**Affiliations:** a Leibniz Centre for Agricultural Landscape Research (ZALF), Müncheberg, Germany; b Department for Geography, Philipps-University of Marburg, Germany; c Julius Kühn Institute (JKI) – Federal Research Centre for Cultivated Plants, Institute for Crop and Soil Science, Braunschweig, Germany

**Keywords:** Rainfall simulation, Water erosion, Measurement techniques, Review of the characteristics of rainfall simulators in soil erosion research studies.

## Abstract

Rainfall simulators are widely employed in soil erosion studies, and it is common for these simulators to be customized to address specific research questions. Nevertheless, there are certain characteristics that rainfall simulators should fulfill in the context of soil erosion studies. Rainfall simulators should simulate natural precipitation as accurately as possible. It is essential to monitor the size spectrum of generated raindrops, their maximum or terminal velocity, the uniformity of the surface distribution of rain, the kinetic energy and the overall intensity of the rain. This review aims to outline the characteristics and the corresponding measurement methods for rainfall simulators in soil erosion research. Electronic instruments like distrometers are considered more suitable for precise and comprehensive measurements than traditional instroments or literature based derivatives. By adhering to these characteristics, researchers can ensure the reliability and accuracy of their findings. Consequently, this overview serves as a valuable resource for researchers seeking to employ rainfall simulators in their investigations of soil erosion.

## Review Highlights

• Characteristics of rainfall simulators in soil erosion research studies.
• Measurement techniques of these characteristics.
• Factors of the experimental question to be considered before designing a rainfall simulator.

## Graphical abstract

**Figure:**
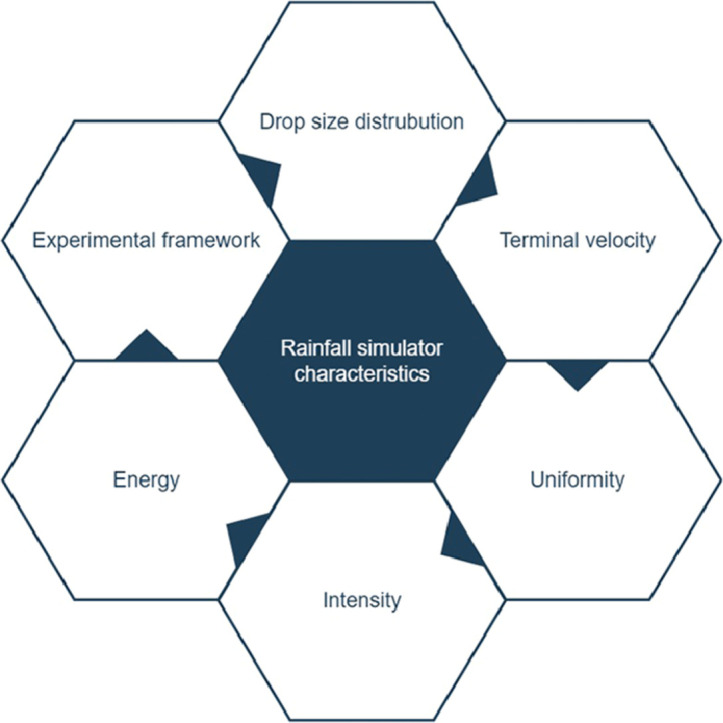

Specifications table

**Table:** 

Subject area:	Agricultural and Biological Sciences
More specific subject area:	*Soil science*
Name of the reviewed methodology:	*Artificial rainfall simulation*
Keywords:	*Rainfall simulation, Water erosion, Measurement techniques*
Resource availability:	*NA.*
Review question:	*What are the key characteristics of rain simulators for soil erosion studies?*

## Method details

Characteristics of rainfall simulators in soil erosion research studies:

The primary purpose of rainfall simulators is to investigate the interaction between rainfall and the land surface, including processes like soil erosion, infiltration, and runoff [1]. Rainfall simulators are designed to simulate rainfall adequately. Moreover, producing rainfall with varying rainfall characteristics allows for a better understanding of the erosive potential of rainfall, which is defined as the ability of rainfall to cause soil erosion [2]. Early studies by Hermsmeier [3], Meyer and Harmon [4], and Bubenzer and Jones [5] identified several key factors crucial for rainfall simulators used in soil erosion research. These factors encompass the (natural) drop size distribution, terminal velocity of individual raindrops, rainfall uniformity, rainfall intensity and the energy of the produced rain. For the European region, studies conducted by Kainz et al. [6], Auerswald et al. [7], Iserloh et al. [8], and Kavka et al. [9] have contributed significantly to the comparison of rainfall simulator characteristics. It is essential to acknowledge that various rainfall simulators with varying rain reproduction and construction characteristics exist. These simulators have been tailored to fulfill specific scientific tasks, making standardization impractical.

Generally, rain simulators can be categorized into two groups: field (portable) simulators and laboratory (non-portable) simulators. Field simulators have been discussed in works by e.g., Meyer and Harmon [4], Cerdá [10], Kainz et al. [6], Auerswald et al. [7], Abd Elbasit et al. [1], Iserloh et al. [8], Chifflard et al. [11], Newesely et al. [12] and Wilson et al. [13]. Laboratory simulators have been explored in the works of, e.g., Mhaske et al. [14], Kavian et al. [15], Bahddou et al. [16], and Lassu et al. [17]. Furthermore, rain simulators can be classified based on the method employed for drop formation: gravitational (dropping) and pressurized simulators [18]. Dropping simulators produce raindrops through free-fall after exiting the simulator tubes [19]. Due to the needed height to achieve terminal velocity of the drop, this kind of rainfall simulator is often used in laboratory experiments [8]. Pressurized simulators can be further categorized into two groups: those directing the stream upwards, where drops are formed gravitationally, and those directing the stream downwards [19]. Pressurized simulators are often used in field experiments. Due to the lack of standards, researchers often find themselves compelled to devise their own designs to suit their particular needs [13].

This review aims to give an overview of the characteristics of rainfall simulators for soil erosion research, with a particular focus on the methods used to capture these characteristics. This review will serve as an overview of the design of customized rainfall simulators and as a guide for the discussion of rainfall simulator results. The review is structured such that each of the aforementioned key characteristics is briefly discussed and measurement technics are explained.

### (Natural) Drop size distribution

The realistic representation of the (natural) drop size distribution (DSD) is crucial in rainfall simulators to emulate rainfall conditions in a given region [20]. The DSD is defined as the mean number of raindrops within a specific diameter range per unit volume of air (mm^−1^ *m*^−3^). During a natural rainstorm, the DSD is statistically distributed, and several distribution models have been proposed in the literature to represent the DSD based on observed data, including the gamma distribution [21] or log-normal distribution [22]. One of the most widely used distributions is the negative exponential model, introduced by Marshall and Palmer [23]. It assumes an exponential decrease in the number of drops as their size increases. Early investigations in the USA, such as by Laws and Parsons [24], extensively examined the natural DSD. It was found that the rain intensity (mm *h*^−1^) influenced the DSD and vice versa. For the European region, data collection occurred at a later stage. Cerdá [10] determined drop sizes for the Mediterranean region under different rain intensities ranging from 1.5 mm to 2 mm. The German Weather Service (DWD) has determined mean drop size values for Germany, ranging between 1 mm to 2 mm [25]. DSD varies depending on the climate zones but also on weather patterns, e.g., the intensity of the rainfall [20,26], different cloud types [27], and further seasonal weather effects, like e.g. temperature of the rain, evaporation and size sorting by wind effects [28]. The collected data provided insights into the typical DSD for different regions and rainfall intensities. This theoretical background of the DSD enabled the development of rainfall simulators for soil erosion studies. Kromer et al. [29] summarized the drop size values for some German rainfall simulators, which range from 1.9 to 2.9 mm. Iserloh et al. [8] summarize the drop size values for some European rainfall simulators, ranging from 0.5 mm to 6.5 mm.

However, it is important to note that the actual DSD in rainfall simulations can vary depending on factors such as the drop generation method, the setup of the rainfall simulator, and the intended research objectives [30]. Rainfall simulators employ different techniques, such as nozzles, sprayers, or specialized equipment, to produce raindrops of desired sizes [8]. In recent studies, a determination of DSD is often neglected because already-known nozzles are used. However, if an unknown nozzle or technique is used, numerous procedures have been developed to measure DSD in rainfall simulation studies. These can be divided into traditional methods, involving manual sampling or visual observation, and new methods, which use techniques such as laser diffraction for more efficient and precise measurements. Traditional methods are based on established practices that are usually inexpensive and easy to apply. These include:

*The stain method* - The stain method operates based on the principle that when a raindrop strikes a uniform absorbent surface, the resulting stain is directly related to the diameter of the drop [31]. Various absorbent surfaces are suitable for this method, including filter paper, blotting paper, blueprint paper, paper toweling, photographic paper, machine tape, and glazed paper [32]. For a short period, rain drops are allowed to land on the absorbent surface and are then analyzed. Cerdá [10] used filter paper, coated with a water-soluble dye so that the drops left circular stains on impact. Kathiravelu et al. [30] reported that several iterations of this method improved the measurement accuracy over time. However, it is important to note that this method is susceptible to potential inaccuracies caused by the splashing of large raindrops upon impact [32]. In the analysis stage, the collection surface is often scanned or photographed, and digital image processing techniques are applied to extract the size and position of the stains. Advanced image processing algorithms can be utilized to segment and measure individual stains, enabling the determination of DSD [33].

*The flour method* - In this method, raindrops are collected in a container filled with flour. Afterwards, the flour is dried, and the pellets are sieved. In the originally developed method by Bentley [34], pans of flour ten inches in diameter and one inch deep, with a smooth, uncompact surface, were exposed to the rainfall for intervals between seconds and minutes, depending upon the rain intensity. Later studies have used different versions of the flour method to analyze DSD successfully [30]. However, the basic procedures remained the same. The test duration should be short to avoid duplicate drop counts [30]. This straightforward approach continues to be utilized as a cost-effective method that does not require expensive equipment, as shown in studies such as Ngezahayo et al. [35] or Živanović et al. [18]. Mazon and Vinas [36] suggest this method for experimental use in schools and demonstrations to farmers as it is easy to apply and understand. However, it is reported, that this method slightly overestimates the raindrop diameter size [37] and requires a high sampling number [30].

*Image processing method* - Photography involves the utilization of a camera to capture images of raindrops, and this approach has been shown to yield highly accurate results, as exemplified by Tullis [38]. Nevertheless, a potential source of error in this method is the determination of the distance between the raindrop and the camera lens, which relies on a single focal point [37]. Moreover, digital pixilation and the time consuming nature of the photographic technique were found to limit their practical use [30]. Light infiltration problems have restricted the use of the photographic method to laboratory analyses, as shown for example by Kavian et al. [15].

*The oil method* – introduced by Eigel and Moore [32]. This method relies on the principle that raindrops immersed in a less dense and more viscous fluid, which prevents evaporation and condensation, allowing drop counting and accurate size measurement [39]. In the oil method, a specific oil mixture is prepared, typically a combination of motor and heavy mineral oil in a 2:1 ratio as suggest by Eigel and Moore [32]. Further viscose liquids, such as Vaseline or a hydraulic fluid mixture are reported by Kathiravelu [30]. During a rainfall event, raindrops fall into the oil mixture and become immersed. The viscosity and hydrophobic nature of the oil helps to preserve the shape of the raindrops, allowing to analyze the size and shape via photograph or microscope. The recorded photograph is then analyzed using an image processing software or measurement techniques to determine the DSD [40,41]. The oil method offers a practical and effective approach for measuring DSD, especially in controlled laboratory or experimental settings, but is not suitable for field applications [42].

*Electronic and automated instruments* - as new methods for DSD measurement. The traditional described methods to measure DSD are not able to measure continuously during a rainfall experiment, are inconvenient to operate [43], and cannot measure more than one drop size simultaneously [37]. To overcome these shortcomings, various instruments, based on different principles, have been developed to directly and continuously measure DSD, as well as other rainfall characteristics like for example rainfall intensity velocity and kinetic energy, constituting a step forward towards a complete characterization of precipitation with one instrument [41]. One of the pioneering instruments in this field is the Joss-Waldvogel distrometer [44], which was the first automatic device capable of measuring the sizes of raindrops based on the vertical component of raindrop momentum [45]. However, the Thies Clima Laser Precipitation Monitor (Thies GmbH, Göttingen, Germany) or the OTT Parsivel distrometer (OTT Hydrometer, Loveland, Colorado, USA) are also frequently used in calibrating DSD for rainfall simulators in soil erosion studies [28,46]. Another approach involves the use of piezoelectric transducers (PTs) that leverage acoustic principles to measure DSD and kinetic energy of raindrops [1,47]. Another advantage of these new methods is the ability to take measurements in real-time during the experiment instead of relying solely on pre-experiment calibration and providing a more comprehensive understanding of the evolving droplet sizes and distribution throughout the entire experimental duration. Shen et al. [48] and Tullis [38] compared some of the so far used electronic instruments, which can be classified in impact distrometers [38] and optical distrometers [35] and concluded that the application of impact distrometers can be problematic during experiments in combination with wind. Optical distrometers are limited to counting droplet totals because optical distrometers separate raindrops into different drop-size and velocity classes and filter droplets out that do not fit into the appropriate classes [30].

In summary, knowing the DSD is essential for rainfall simulators as it provides insights into various aspects of rainfall characteristics, such as the kinetic energy, and thus the erosivity of the produced rain [49]. Accurate representation of the DSD ensures that the simulated rainfall closely matches the characteristics of natural rainfall. Traditional, inexpensive, and rapid measurement of the DSD are available but may have limitations in accuracy and detail. For precise and comprehensive measurements of the DSD, electronic instruments, like distrometers and PTs are considered more suitable and are widely employed.

### Terminal velocity

Terminal velocity is defined as the maximum velocity at which a falling raindrop can descend under the influence of gravity and air [50]. Raindrops reach their terminal velocity when falling from a certain height, where the sum of the drag force and the force of gravity acting on the raindrop becomes equal [41]. Terminal velocity varies with the size and shape of raindrops and atmospheric conditions, such as air density [42]. In rainfall simulator studies, the terminal velocity of raindrops is a crucial parameter as it determines the produced energy of the rainfall during the experiment. The terminal velocity of individual raindrops can be measured directly or indirectly. As a direct measurement method, optical distrometers [48] or photometry [15] are frequently used and described in detail by, e.g., Lanza et al. [41].

**Fig. 1:**
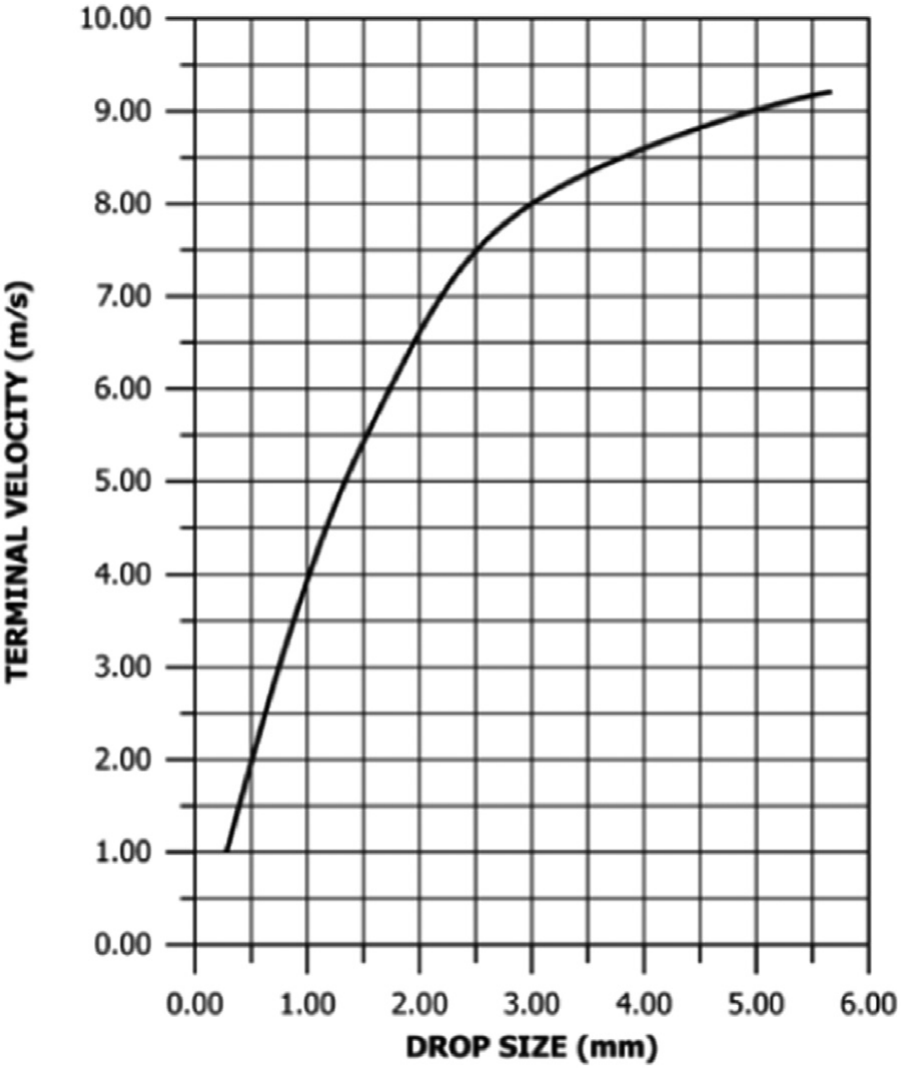
Drop size to terminal velocity correlation according to Etheridge [37].

Laws [51] conducted an early study focusing on the fall velocities of raindrops, revealing that terminal velocity decreases with smaller droplets. Subsequently, Laws and Parsons [24] established a relationship between drop size and fall velocity (Fig. 1), widely used as an indirect method to estimate the velocity of individual raindrops. Fall height in rainfall simulators directly influences the terminal velocity raindrops can achieve during the simulated rainfall event. Pressurized nozzle simulators have demonstrated an advantage over non-pressurized simulators, as the latter may require fall heights of 10 m or more to attain terminal velocity [52]. While indirect measurement methods simplify raindrop velocity determination by relying on drop size, errors in drop size determination can lead to compounded errors in fall velocity [37]. In addition, determining accurate fall velocities in outdoor experiments poses challenges due to the influence of wind and the resulting acceleration of raindrops [53,54]. This complication makes it “difficult” or even “impossible” to precisely measure the fall velocity during outdoor experiments [8].

In summary, terminal velocity of the produced rain drops can be measured simultaneously with the DSD via a distrometer or photography. Moreover, the relationship between DSD and velocity is frequently used to estimate the rain drop velocity in rainfall simulation studies but poses the risk of compounded errors and simplify the terminal velocity as a function of drop size.

### Uniformity

The uniform distribution of rainfall across the target area and for a specified time is a crucial aspect of rain simulation. The Christiansen Uniformity Coefficient (CU), proposed by Christiansen in 1942, has emerged as the most commonly utilized metric.

(1) $$CU=100\%\left(1-\;\frac{\sum_{i=1}^{n}\left|x_{i}-x_{avg}\right|}{n*x_{avg}}\right)$$

where, CU = Uniformity of spray, (%)

x_i_ = is individual water amount per rain collector, (ml)
x_avg_ is the arithmetic mean of applied water amount per rain collector, (ml)
n is the total number of rain collectors.

**Fig. 2:**
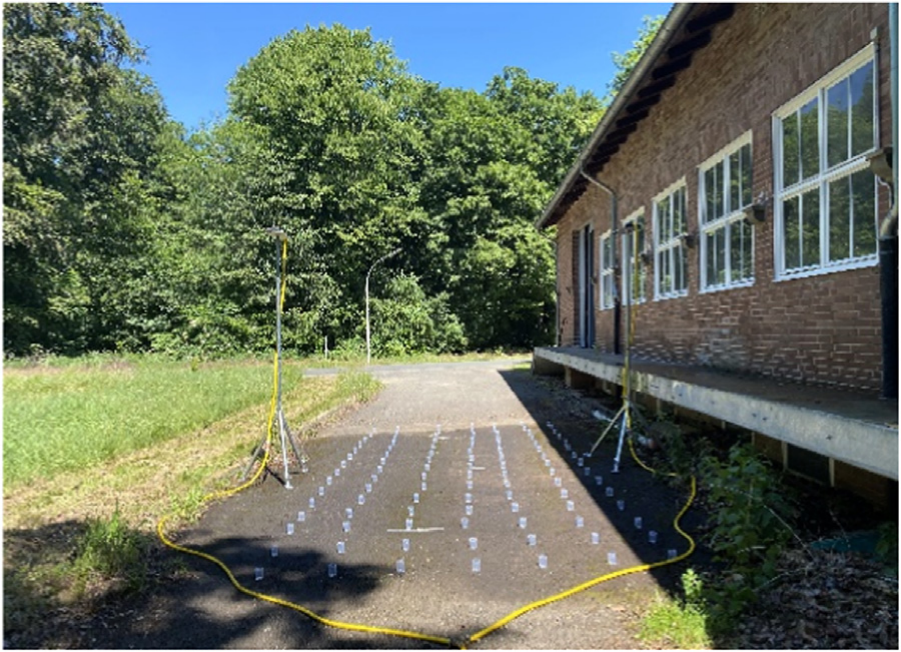
Calibrating procedure for spatial uniformity testing needed for CU coefficient. Own study.

It is calculated by comparing the observed coefficient of variation (CV) of measured rainfall depths with the ideal CV that represents a perfectly uniform distribution of rainfall [55]. A higher CU value indicates a more uniform distribution of rainfall, with a maximum value of 100 representing perfect uniformity. Iserloh et al. [8] reported CU values for European rainfall simulators ranging from 76% to 97% at various pressures and heights. Nonetheless, it is essential to consider uniformity when calibrating rainfall simulators, e.g. overlapping rainfall can result in areas with increased or decreased rainfall intensity and subsequent effects on soil erosion [37]. Moreover, a well-distributed droplet pattern is crucial for accurately measuring infiltration in the field, which is particularly significant for parameterizing soil erosion models [5]. To improve the uniformity, oscillating or pivoting movements are often incorporated into the rainfall simulator, like for example in the Norton rainfall simulator [42]. Also fans [56] or manual movements are applied to achieve best uniformity. Measurement methods for the CU are relatively simple and straightforward. Humphry et al. [57] employed cups with a diameter of 100 mm placed at defined distances from each other to collect rainfall (Fig. 2). The rainfall was collected over a defined period of time. The cups were subsequently weighed to determine the volume of precipitation. However, it is worth noting that while the CU has been widely adopted, there are alternative methods, for example, distribution uniformity (DU), application efficiency (Ea) or deep percolation (DP) available for assessing rainfall uniformity in rainfall simulator experiments [58] because the experimental resolution (including the number, density, and spatial configuration of collection containers) and external factor such as wind or slope inclination can impact the calculated CU [55]. Consequently, defining a minimum value for an acceptable CU value is difficult. Instead, Green and Pattison [55] recommended that conducting multiple assessments of CU at different experimental resolutions is important.

### Intensity

The intensity of a rainfall simulator is relatively easy to characterize. A standard pluviometer (e.g. Hellman rain gauge) is frequently used for this purpose, but also the collection of the total runoff from a known plot area is used [37]. Rainfall intensity and CU can be determined simultaneously by using rain gauges. When designing a rainfall simulator, it is important to define the required intensity of planned simulations beforehand. Intensities can be adapted according to typical rainfall events of climatic regions. In central Europe, the intensity rarely exceeds values of 75 mm/h [20]. Various studies have utilized different rainfall simulators to achieve specific intensities. The Swanson-type rainfall simulator, used for parameterizing the physical soil erosion model WEPP and the German rainfall simulators reported in Auerswald et al. [7], typically apply an intensity of approximately 60 mm/h. This corresponds to the intensity of a 5-year extreme event. Newesely et al. [12] reported an average intensity of 100 mm/h for the Innsbruck rainfall simulator, with certain areas within the 10 m^2^ plot experiencing intensities of up to 140 mm/h. Similar values are reported in Etheridge [37] for the Auburn University rainfall simulator. To mitigate unwanted high intensity levels during rainfall simulations, a commonly employed technique involves brief interruptions in the rain using discs or tarpaulins, lasting anywhere from 1 to 5 s or to inject air into the water stream [19]. Generally, it should be aimed to achieve a high uniformity of the rainfall intensity across the plot.

### Energy

To detach soil aggregates from the soil surface requires energy expenditure [59]. The primary energy source in water erosion processes is attributed to the rainfall kinetic energy (KE). Mathematically, KE is defined as half of the product of the drop's mass and velocity squared and is expressed in joules [1]. In soil erosion research studies, KE can be specified as volume-specific rainfall KE (J *m*^−2^ mm^−1^) or time-specific rainfall KE (J *m*^−2^ *h*^−1^) generated by raindrops through their impact on the soil surface [20,60]. Furthermore, KE is widely used as an indicator of rain erosivity [49,60]. As rain comprises a range of drop sizes, the KE is influenced by the nature of the distribution of these sizes [20]. In particular, larger drops have greater mass and greater terminal velocity such that a disproportionate amount of the total KE results from the larger drops. However, the interaction of rain with the soil surface is complex, and it is not solely determined by KE. The amount of soil dislodged is also influenced by external factors such as micro relief, slope, soil type, soil structure, moisture content, wind, and other factors [20]. Various methods have been used to measure rainfall KE. These can be classified into direct and indirect methods. Abd Elbasit et al. [1] reported the use of a transducer as a direct method to measure rainfall KE. Another direct method is the use of optical distrometers, measuring drop size and velocity simultaneously, allowing the calculation of KE from these measured parameters. Iserloh et al. [8] used the equation from Fornis et al. [61] to determine the KE as volume-specific rainfall KE (J *m*^−2^ mm^−1^) from measured distrometer data, which is defined as:

(2) $$KE=\left(\frac{KE_{R}}{I}\right)$$

Where I is the rainfall intensity (mm *h*^−1^) obtained via a distrometer and KE_R_ is the rate of kinetic energy expenditure (J *m*^−2^ *h*^−1^) for every 1-min period, which is defined as:

(3) $$KE_{R}=\left(\frac{\pi }{12}\right)\left(\frac{1}{10^{6}}\right)\left(\frac{3600}{t}\right)\left(\frac{1}{A}\right)\sum_{i=1}^{20}n_{i}D_{i}^{3}\left(v_{D1}\right)2$$

Where A is the sampling area of the distrometer (m²), n_i_ the number of drops of diameter D_i_ (mm);v_Di_ is the measured fall velocity (m/s) of drop with diameter D_i_ and t is the time interval of 60 s.

Rainfall KE can be estimated indirectly by measuring the DSD and empirical raindrop fall velocity, as shown under points 1 and 2 of this review. KE is obtained by summing the energy of each drop size group multiplied by the percentage of energy of the according group [37]. However, by calculating KE for rain drops, a strong correlation between the KE and rainfall intensity (I) (*mm h*^*-1*^) was observed when rain drops are travelling at or near their terminal velocity [20,59]. Therefore, disregarding the effects of wind velocity, the relationship between I and KE provides another method to estimate rainfall KE [60]. Consequently, various KE-I relationships have been established based on empirical observations (some examples can be found in Table 1) [43]. Originally, this method is mainly used for natural rainfall, as empirical values are used. However, its application in rainfall simulation studies has also been reported [56].

**Table 1:** Most common relationships between kinetic energy (KE) (J *m*^−2^*h*^−1^) and rainfall intensity (I) (mm *h*^−1^).

Reference	Equation
Wischmeier and Smith [2]	$KE=I\left(11.9+8.73logI\right),\;I\leq 76mm\;h^{-1}$ $KE=28.3I,\;I>76mm\;h^{-1}$
Brown and Foster [62]	$KE=29I(1-0.72e^{-0.05I})$
McGregor et al. [63]	$KE=29I(1-0.72e^{-0.082I})$
van Dijk et al. [49]	$KE=28.3I(1-0.52e^{-0.042I})$

There are sources of uncertainty reported when using indirect methods or literature relationships [1] because KE is then based on the assumption that diameters or velocities from natural rainfall apply to simulated rainfall. While the importance of understanding the KE of rainfall simulator studies is widely acknowledged, direct measurements are rare because these are time consuming and expensive.

Iserloh et al. [8] found that KE values generated by European rainfall simulators are lower than those reported in the literature for natural rainfall. This was primarily due to the limited fall height produced by the simulators, which prevents large raindrops from reaching their terminal velocity [8]. Additionally, the spatial patterns of the KE inside the plot can vary substantially, as shown by Lassu et al. [17] for the Wageningen rainfall simulator. The spatial distribution of the KE within a plot area depends on the uniformity achieved. However, the energy that affects the soil is the only real factor that connects natural rain and simulated rain with the soil erosion process [42].

In summary, KE is a crucial factor in rainfall simulator studies because KE represents an indicator for the erosivity of the simulated rainfall [1]. Thus, KE is critical to the soil erosion process and subsequent processes such as runoff and nutrient loss. Direct measurements of KE are costly but precise. Indirect calculations of KE require knowledge of the DSD, intensity and measured terminal fall velocity or empirical laws linking terminal velocity and drop sizes. However, indirect calculations are widely employed and reported in rainfall simulation studies.

### Plot size

The plot size used in rainfall simulations is a critical factor influencing the accuracy and representativeness of the results. Small field rainfall simulators offer several advantages, including their cost-effectiveness, ease of transport in remote or inaccessible areas, and low water consumption [8]. Over several decades, more than 100 rainfall simulators with plot dimensions typically less than 5 m^2^ have been developed [8]. They are suitable for investigating specific research questions or evaluating soil erosion processes at a local scale. For example, smaller plot sizes are commonly employed to assess runoff activation and the initial phase of the soil aggregates detachment process and estimate infiltration rates [9]. Moreover, their compact dimensions and structure make small field rainfall simulators suitable for use on steeper slopes. On the other hand, larger plot sizes are employed in field-scale experiments. These larger plots provide a more realistic representation of natural or tillage conditions and allow for the examining complex interactions between rainfall, soil, and vegetation [20]. However, larger field rainfall simulators require more extensive resources and logistical considerations. The choice of plot size influences the selection and design of rainfall simulators. Smaller plots can be adequately covered by smaller portable rainfall simulators, while larger plots may necessitate the use of stationary or mobile simulators with greater water delivery capacity. Laboratory rainfall simulators are also used on rather small plot sizes, as the investigations are usually limited to individual plants or small soil covered test areas. Kinnell [64] reviewed the requirements of plot designs in soil erosion studies. The rectangular shape appears to be the most suitable plot shape [64]. Nevertheless, plot size, length, shape, and plot surface characteristics (e.g. soil type, slope, soil moisture) should be mandatory information in any publication.

### Transportability

**Fig. 3:**
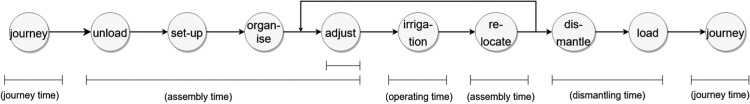
Process stages of rainfall simulation. Modified according to [29].

Rainfall simulators are designed with specific research questions in mind, catering to either field or laboratory settings, or sometimes both. For instance, in field trials investigating undisturbed soils, the emphasis lies on mobility and efficiency in terms of labor and time. Kainz et al. [6] reported up to 10 h of assembling times for some of the tested German field rainfall simulators. This is considered too much for a field rainfall simulator in order to conduct experiments with proper replications [8]. Similarly, the number of available workers should be considered, keeping in mind that field rainfall simulations can be discontinuous due to changing weather conditions [6]. Furthermore, limitations in water provision and logistics will set boundary conditions of field rainfall simulators. Small-scale field rainfall simulators exhibit reduced water requirements and are generally easier to transport but might limit the research questions which can be answered. Additional boundary conditions are given by the logistics, e.g., the need for tractors, energy, and fuel (Fig. 3). Consequently, a detailed transport plan should be elaborated in advance for field rainfall simulators. On the other hand, laboratory setups offer a fixed arrangement that reduces the need for frequent modifications and allows for more intricate experiments to be conducted. The fixed setup of equipment minimizes the logistic processes. Laboratory setups are particularly beneficial for conducting experiments requiring precise control over variables and environmental conditions.

### Reproducibility

Reproducibility is a critical aspect of rainfall simulator studies to ensure the reliability and validity of the experimental results. In this context, standardized setups, detailed experiment documentation, replication and randomizations in experimental designs and data analyses are crucial. The cost factor per plot associated with rainfall simulations, especially for field rainfall simulators, is a critical consideration that has been largely neglected and not thoroughly analyzed in many studies. Kromer et al. [29] described that the total costs per plot, which depend significantly on the plot size and process costs, like water and fuel demand, are between 200 and 1000 EUR for their investigated German rainfall simulators. The reproducibility of field rainfall simulators relies not only on the factors above but also on natural conditions, including weather conditions, particularly wind, and soil and vegetation properties of the plot itself.

Reproducibility is certainly one of the main advantages of laboratory rainfall simulators, as a stable and controlled environment can be created. Moreover, for laboratory rainfall simulators, there has been a notable shift in the development towards fully automated systems [9]. These advanced computer-controlled simulators enable the simulation of rainfall events with variable intensity and kinetic energy over time [65]. This technological advancement enhances the accuracy and realism of the simulated rainfall and simplifies the experimental process.

## Conclusion

Developing a universal rainfall simulator for soil erosion studies suitable for all experimental conditions and scientific purposes is not achievable. Instead, rainfall simulators must be tailored to specific research objectives, acknowledging that boundary conditions can constrain rainfall simulators substantially. Several factors must be carefully taken into account during the design process. Firstly, the research question at hand and the intended application of the simulator must be well-defined. Different research objectives demand varying degrees of precision and control, making it necessary to customize the simulator accordingly. Secondly, the simulator's capability to replicate desired weather conditions, DSD, and intensity becomes crucial. Related to this, determining the erosive energy to be achieved is equally important. A comprehensive understanding of the regional conditions is essential to ensure accurate simulation. If the rainfall simulator is intended for field use, additional considerations relating to infrastructure are vital. Practical aspects, such as ease of setup, transportability, water supply, and reproducibility, influence possible plot sizes and rainfall simulator characteristics. Available resources during the experiment, such as material, workmanship, and technology, should be taken into account during the construction phase. Thoroughly considering these factors will lead to a well-designed rainfall simulator for the intended purpose and help answer the research question on hand.

## CRediT authorship contribution statement

**Tobias Koch:** Conceptualization, Investigation, Writing – original draft. **Peter Chifflard:** Supervision. **Peter Aartsma:** Writing – review & editing. **Kerstin Panten:** Supervision, Writing – review & editing.

## Declaration of Competing Interest

The authors declare that they have no known competing financial interests or personal relationships that could have appeared to influence the work reported in this paper.

## Data Availability

No data was used for the research described in the article.
